# Supplementary material for: Association between oral microbiome and five types of respiratory infections: a two-sample Mendelian randomization study in east Asian population
Source: Front Microbiol. 2024 Apr 10;15:1392473. doi: 10.3389/fmicb.2024.1392473 (PMC11039966; doi:10.3389/fmicb.2024.1392473)
Supplement: Supplementary file 1 [file Data_Sheet_1.zip › Supplementary Materials/MR plots of saliva/Chronic sinusitis/s__Lachnoanaerobaculum_sp000287675_mgs_978/leave_one_out.pdf]

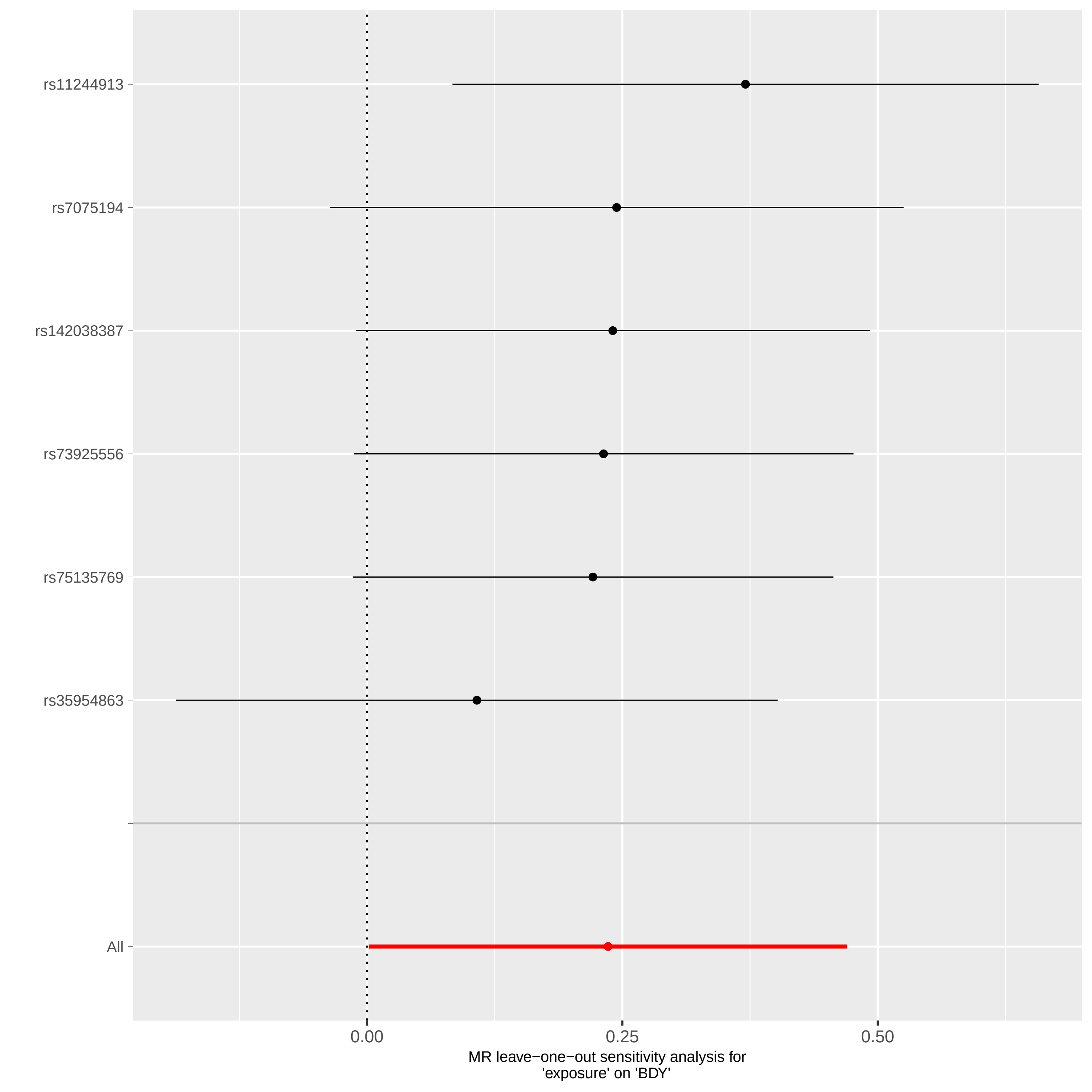

rs11244913
rs7075194
rs142038387
rs73925556
rs75135769
rs35954863
All
0.00
0.25
0.50
MR leave−one−out sensitivity analysis for
'exposure' on 'BDY'
